# Tailoring the Best Positive End-Expiratory Pressure Through Invasive Right Ventricular Pressure-Volume Loops in a Patient Supported by Veno-Arterial Extracorporeal Membrane Oxygenation

**DOI:** 10.1097/MAT.0000000000002238

**Published:** 2024-05-22

**Authors:** Ilaria Protti, Antoon van den Enden, Paolo Meani, Maarten ter Horst, Nicolas M. Van Mieghem, Christiaan L. Meuwese

**Affiliations:** From the * Department of Cardiology, Thorax Center, Cardiovascular Institute, Erasmus Medical Center, Rotterdam, The Netherlands; † Department of Intensive Care for Adults, Erasmus Medical Center, Rotterdam, The Netherlands; ‡ Department of Pathophysiology and Transplantation, Università degli Studi di Milano, Milan, Italy; § Department of Cardiothoracic Surgery, Heart and Vascular Centre, Maastricht University Medical Centre, Maastricht, The Netherlands; ¶ Faculty of Health, Medicine and Life Sciences, Maastricht University, Maastricht, The Netherlands; ‖ Thoracic Research Center, Innovative Medical Forum, Collegium Medicum Nicolaus Copernicus University, Bydgoszcz, Poland; # Department of Anesthesiology, Erasmus University Medical Center, Rotterdam, The Netherlands.

**Keywords:** cardiac physiology, extracorporeal membrane oxygenation, mechanical ventilation, pressure-volume loops, right ventricular function

## Abstract

Patients undergoing veno-arterial extracorporeal membrane oxygenation (VA-ECMO) typically suffer from cardiogenic pulmonary edema and lung atelectasis, which can exacerbate right ventricular (RV) dysfunction through an increase in lung elastance and RV afterload. Invasive mechanical ventilation settings, and positive end-expiratory pressure (PEEP) in particular, can help to improve RV performance by optimizing lung recruitment and minimizing alveolar overdistention. In this report, we present a VA-ECMO supported patient in whom *in vivo* RV pressure-volume (PV) loops were measured during a decremental PEEP trial, leading to the identification of an optimum PEEP level from a cardio-respiratory viewpoint. This innovative approach of tailoring mechanical ventilation settings according to cardio-respiratory physiology through *in vivo* RV PV loops may provide a novel way to optimize hemodynamics and patient outcomes.

Veno-arterial extracorporeal membrane oxygenation (VA-ECMO) is a rescue therapy for patients with life threatening cardiopulmonary collapse. In this setting, cardiogenic pulmonary edema and lung atelectasis often occur, leading to an increase in lung elastance and work of breathing and, therefore, causing dramatic oxygen consumption.^1^ Moreover, extra-alveolar capillary vessels become increasingly compressed by collapsing lung units, resulting in higher pulmonary vascular resistance (PVR), which ultimately represents the main determinant of right ventricular (RV) afterload.^2^

Positive end-expiratory pressure (PEEP) affects RV afterload by modifying the degree of lung recruitment and alveolar overdistention.^2^ Thus, optimizing the management of invasive mechanical ventilation might improve patients’ hemodynamics, thereby promoting overall cardiac recovery and weaning from VA-ECMO support.

To date, there is still a significant knowledge gap in literature concerning the effects of positive pressure ventilation on hemodynamics, especially in the context of VA-ECMO. Therefore, we present a case of a patient supported with VA-ECMO for refractory cardiac arrest, in which the best PEEP from a cardio-respiratory perspective was successfully set through the application of invasive RV pressure-volume (PV) loops.

## Case Report

A 62 year old man (Body Mass Index (BMI) = 27 kg/m^2^), with a medical history of hypertension and ischemic cardiomyopathy (ejection fraction 30%), suffered a witnessed cardiac arrest on basis of a shockable rhythm. After approximately 14 minutes of cardiopulmonary resuscitation and seven defibrillations, return of spontaneous circulation occurred after which the patient was admitted to the intensive care of our institution. Veno-arterial extracorporeal membrane oxygenation support was started during a second in-hospital cardiac arrest due to pulseless ventricular tachycardia.

**Figure 1.**
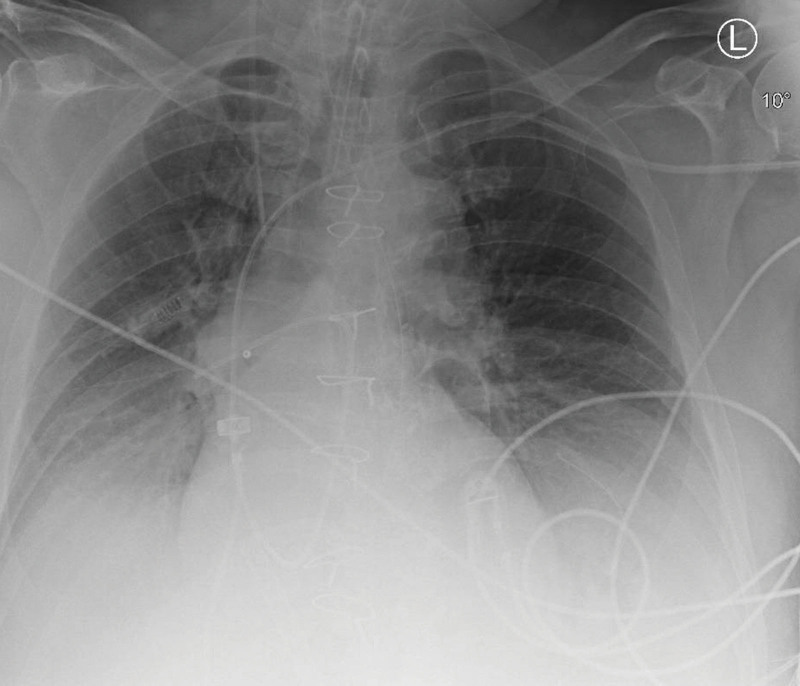
A chest x-ray taken on the day of the measurements, at 10 cm H_2_O of PEEP. PEEP, positive end-expiratory pressure.

Coronary angiography suggested a spontaneous coronary dissection of the distal LAD, which was left untreated because of TIMI 3 blood flow. An x-ray of the chest showed extensive atelectasis of the dependent lung regions combined with cardiogenic pulmonary edema (Figure 1), initially managed with a PEEP of 10 cm H_2_O, associated with a Plateau Pressure of 25 cm H_2_O and a driving pressure of 15 cm H_2_O. At day 2 of ECMO support, intravenous diuretics (furosemide) were initiated after hemodynamic stabilization had occurred.

**Figure 2.**
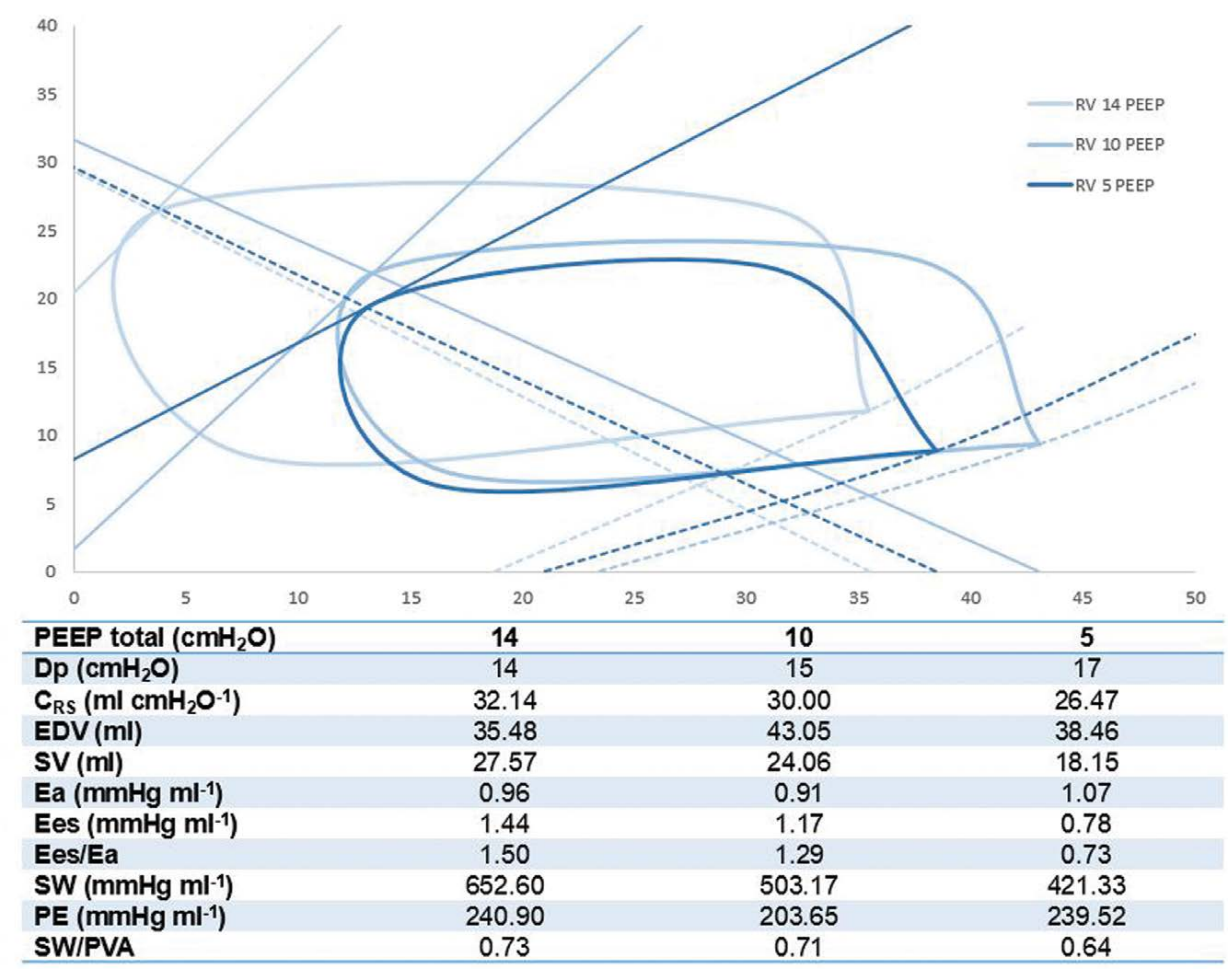
Changes in RV PV loops and respiratory system mechanics at different PEEP levels in a patient supported by 3.5 L/minute of VA-ECMO blood flow. C_RS_, respiratory system compliance; Dp, driving pressure; E_a_, arterial elastance; EDV, end-diastolic volume; E_es_, ventricular end-systolic elastance; E_es_/E_a_, ventriculo-arterial coupling; PE, potential energy; PEEP, positive end-expiratory pressure; SV, stroke volume; SW, stroke work; SW/PVA, stroke work/pressure-volume area ratio. VA-ECMO, veno-arterial extracorporeal membrane oxygenation.

After 4 days of VA-ECMO support, a weaning trial in combination with invasive PV loops based on conductance catheter measurements was performed in the context of a prospective study (NCT05909280). Right ventricular PV loops^3^ were also recorded during a decremental PEEP trial (14-10-5 cm H_2_O) while the patient was sedated and paralyzed, ECMO blood flow was 3.5 L/minute (1.7 L/minute/Body Surface Area (BSA)) and the ventilator was set in a volume controlled mode with tidal volumes of 450 ml (6 ml/kg of ideal body weight), respiratory rate of 12 breaths/minute and FiO_2_ at both natural and membrane lung of 0.65 (Figure 2). Each PEEP level was kept for 5 minutes, vasoactive agents were kept constant (noradrenaline 0.05 mcg/kg/minute, milrinone 0.25 mcg/kg/minute, dobutamine 3 mcg/kg/minute), and no fluid load was administered.

A reduction in PEEP level was associated with an increase in RV afterload (E_a_), a slight increment in RV preload (EDV), and a lower compliance of the respiratory system (C_RS_), RV stroke volume (SV), intrinsic myocardial contractility (E_es_), and ventriculo-arterial coupling (E_es_/E_a_). The combination of a lower RV stroke work (SW) and unchanged potential energy (PE) translated into a loss of mechanical efficiency (SW/PVA) at lower PEEP levels.

Following the decremental PEEP trial, PEEP was kept at 14 cm H_2_O without any further signs of lung derecruitment and successful weaning from VA-ECMO ensued.

## Discussion

In our clinical case, higher PEEP levels were associated with improved RV mechano-energetics. These findings seem best explained by lung derecruitment in the context of extensive alveolar atelectasis, consequently leading to an increased RV afterload likely due to both increased PVR and intrapulmonary shunt (further augmenting hypoxic vasoconstriction). This pressure overload would then explain the acute ventriculo-arterial decoupling but not the reduction in RV E_es_, as the latter represents a load-independent parameter of myocardial contractility. To date, no convincing explanations for this interesting finding have been found in literature, although some authors have proposed the so called “*Anrep effect*” or the *“slow force response”* of myocardial fibers to abrupt afterload induced changes in myocardial stretch.^4^ In the context of an almost unchanged RV preload (since the latter is primarily determined by VA-ECMO blood flow), the reduction in SV itself might be a consequence of the decreased RV contractility. Finally, ventriculo-arterial decoupling together with depression in myocardial contractility led to a reduction of SW alone, ultimately causing a loss in ventricular mechanical efficiency at lower PEEP levels.

Our finding contradicts contemporary conceptions that RV function benefits from low PEEP levels.^5^ Indeed, PEEP is known to reduce systemic venous return^6^ and represents one of the main determinants of RV afterload,^2^ thus possibly leading to a decline in myocardial function.^7^ However, this simplistic view fails to take into account two fundamental arguments: 1) the effect of PEEP on RV function strictly depends on the degree of lung recruitment and alveolar overdistention,^8^ moreover 2) in patients supported by VA-ECMO, the ECMO itself also actively supports and unloads the RV, thus reducing its PV area (PVA) placing the ventricle in a favorable mechano-energetic condition.

In this clinical case, invasive RV PV loops represented an innovative approach to set the best PEEP in a patient supported by VA-ECMO, as they allowed us to optimize both lung recruitment and ventriculo-arterial coupling. In conclusion, tailoring PEEP levels on the basis of cardio-respiratory pathophysiology provides a tool that may help optimize cardiopulmonary status and patient outcomes under VA-ECMO support. In clinical practice, implementation of RV PV loops to optimize ventilatory settings in patients undergoing VA-ECMO requires further research. As the complexity and invasiveness of PV loop measurements likely limit the feasibility of such approach for the purpose of optimum PEEP selection at the bed side, our findings should also be used to validate non-invasive PV loop approaches as based on ultrasound and pulmonary artery catheter measurements.
